# An In-Silico Investigation to Design a Multi-Epitopes Vaccine against Multi-Drug Resistant *Hafnia alvei*

**DOI:** 10.3390/vaccines10071127

**Published:** 2022-07-15

**Authors:** Fahad M. Alshabrmi, Faris Alrumaihi, Sahar Falah Alrasheedi, Wafa Abdullah I. Al-Megrin, Ahmad Almatroudi, Khaled S. Allemailem

**Affiliations:** 1 Department of Medical Laboratories, College of Applied Medical Sciences, Qassim University, Buraydah 51452, Saudi Arabia; fshbrmy@qu.edu.sa (F.M.A.); f_alrumaihi@qu.edu.sa (F.A.); sahar-f-r@hotmail.com (S.F.A.); aamtrody@qu.edu.sa (A.A.); 2 Department of Laboratory and Blood Bank, King Saud Hospital, Unaizah 56437, Saudi Arabia; 3 Department of Biology, College of Science, Princess Nourah bint Abdulrahman University, P.O. Box 84428, Riyadh 11671, Saudi Arabia

**Keywords:** antimicrobial resistance, vaccine, *Hafnia alvei*, molecular dynamics simulations

## Abstract

Antimicrobial resistance has become a significant health issue because of the misuse of antibiotics in our daily lives, resulting in high rates of morbidity and mortality. *Hafnia alvei* is a rod-shaped, Gram-negative and facultative anaerobic bacteria. The medical community has emphasized *H. alvei*’s possible association with gastroenteritis. As of now, there is no licensed vaccine for *H. alvei*, and as such, computer aided vaccine design approaches could be an ideal approach to highlight the potential vaccine epitopes against this bacteria. By using bacterial pan-genome analysis (BPGA), we were able to study the entire proteomes of *H. alvei* with the aim of developing a vaccine. Based on the analysis, 20,370 proteins were identified as core proteins, which were further used in identifying potential vaccine targets based on several vaccine candidacy parameters. The prioritized vaccine targets against the bacteria are; type 1 fimbrial protein, flagellar hook length control protein (FliK), flagellar hook associated protein (FlgK), curli production assembly/transport protein (CsgF), fimbria/pilus outer membrane usher protein, fimbria/pilus outer membrane usher protein, molecular chaperone, flagellar filament capping protein (FliD), TonB-dependent hemoglobin /transferrin/lactoferrin family receptor, Porin (OmpA), flagellar basal body rod protein (FlgF) and flagellar hook-basal body complex protein (FliE). During the epitope prediction phase, different antigenic, immunogenic, non-Allergenic, and non-Toxic epitopes were predicted for the above-mentioned proteins. The selected epitopes were combined to generate a multi-epitope vaccine construct and a cholera toxin B subunit (adjuvant) was added to enhance the vaccine’s antigenicity. Downward analyses of vaccines were performed using a vaccine three-dimensional model. Docking studies have confirmed that the vaccine strongly binds with MHC-I, MHC-II, and TLR-4 immune cell receptors. Additionally, molecular dynamics simulations confirmed that the vaccine epitopes were exposed to nature and to the host immune system and interpreted strong intermolecular binding between the vaccine and receptors. Based on the results of the study, the model vaccine construct seems to have the capacity to produce protective immune responses in the host, making it an attractive candidate for further in vitro and in vivo studies.

## 1. Introduction

Antibiotics are used for treating bacterial infections. An antibiotic is a sedative used to eradicate bacteria or control their growth [1]. Infections caused by viruses cannot be treated by it. Alexander Flemming discovered penicillin in 1928 and it became the first antibiotic. Antibiotic overuse can lead to bacterial infections that are resistant to antibacterial drugs, resulting in antimicrobial resistance (AMR) [2]. Those pathogens that show resistance to antibiotics have a high morbidity and mortality rate [3,4]. Globally ~70,000 total deaths are caused due to AMR each year. Bacteria become resistant to antibiotics when their genetic makeup is changed as a result of over- or underuse of antibiotics. It will take time to develop a vaccine that is safe to use in order to lower the burden of antibiotic resistance [5]. Only good vaccine availability can prevent antibiotic resistance [6,7]. Resistance to vaccines develops extremely rarely because vaccines are given before infection, which means they are prophylactic.

A vaccine is a biological preparation that produces antibodies in the host to stimulate the immune system. Immunity is induced through this process. Edward Jenner developed the first smallpox vaccine using the cowpox virus. Good vaccines are developed by using different approaches that can be categorized into many types. In order to treat anthrax, Louis Pasteur used the bacillus in its weakened form as a vaccine. There was a high number of vaccine experiments conducted after Pasteur in order to prevent several diseases [8]. Using the Pasteur vaccinology rules, Sabin and Stalk created an excellent vaccine for polio. Pasteur vaccinology was used to develop a vaccine against Mycobacterium tuberculosis named Bacillus Calmette Guerin (BCG). However, Pasteur vaccines have some limitations; they cannot be used against pathogens that show constant mutations in their antigens, or against those that cannot be successfully cultured in vitro [9]. Vaccine development is, therefore, expensive and time-consuming. This resulted in a reduced number of culture-based vaccines being developed, and new approaches to developing vaccines being used. Recent advancements in vaccinology have resulted in reverse vaccinology (RV), which is based on a genome database to determine the antigenic surface proteins [10,11,12]. By applying different filters, RV identifies possible vaccine candidates [13]. RV was used to develop the meningococcal serogroup B vaccine (4CMenB). The goal of this method is to identify the reference proteome from subtractive proteomes and predict the B-cell epitopes and T-cell epitopes that will be used as part of constructing good vaccine candidates.

*Hafnia alvei* belongs to the Enterobacteriaceae family of facultatively anaerobic gram-negative bacteria. The role of this genus in human infectious diseases is unknown even though it was first described in 1954. *H. alvei* is the sole member of the Hafnia genus. Its name derives from the Latin name *Hafnia*, meaning Copenhagen [14]. Humans and many animals have *Hafnia* in their gastrointestinal tracts as part of the normal microbiome. Although it can be cultured from a variety of sites, *H. alvei* rarely cause monomicrobial infections. There has been evidence that *H. alvei* can cause human infection on occasion. *H. alvei* is a rare and poorly understood commensal bacterium [15].

Malignancies, trauma, and recent surgeries are among the reasons for *H. alvei* infections. *H. alvei* is naturally resistant or of intermediate susceptibility to amoxicillin–clavulanate, fosfomycin, ampicillin–sulbactam, amoxicillin, narrow-spectrum cephalosporins, tetracycline, and azithromycin [14]. In addition to soil and dairy products, *H. alvei* is found in sewage and human and animal feces. *H. alvei* is a relatively rare cause of infection, having been implicated as a cause of diarrhea, bloodstream infections, meningitis, urinary tract infections, wound infections, intra-abdominal abscesses, and empyema in immune-competent and immunocompromised individuals. It appears that *H. alvei* shares the same antibiotic susceptibility patterns as other Enterobacter species, such as ampicillin resistance and cefazolin resistance [16]. This gram-negative bacterium rarely infects humans, which makes *H. alvei* rare as far as pathogenic bacteria go. Psychotropic bacteria, such as *H. alvei* originate in raw milk and survive in cheese like Camembert. The dominant species in raw milk cheese ripening is *H. alvei* [17].

In this study, a complete proteome of the pathogen *H. alvei* was retrieved from the Uniprot and subsequently the reverse vaccinology pipeline [18,19,20] was applied in order to identify potent vaccine epitopes that can provide substantial immunity against the pathogen. Further, dynamic behavior and stability in the presence of the receptors were confirmed using molecular dynamic simulations [21,22,23]. We believe that in this study, predicted epitopes can provide quite some knowledge for vaccine developers to develop a highly potent and optimized vaccine against *H. alvei* infections.

## 2. Methodology

**Figure 1:**
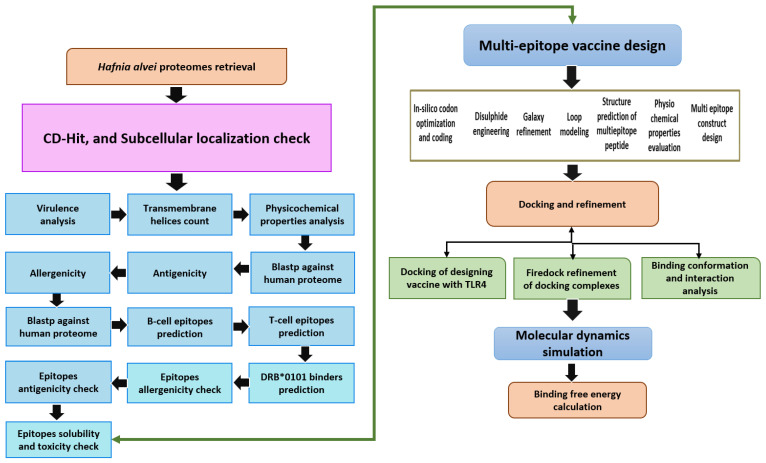
The method used in designing a multiepitope vaccine against *H. alvei* is depicted here. The flow describes subtraction of pathogen proteome through different vaccine parameters, followed by immunoinformatic techniques to map epitopes for the potential vaccine candidates. Finally, biophysics approaches were utilized to investigate vaccine molecule binding and dynamics to immune receptors. The double head arrow indicates that either the epitopes can be used directly in epitope vaccine design or multi-epitopes vaccine design.

According to the flow chart below, a multiepitope vaccine was designed following the methodology described in Figure 1.

### 2.1. Subtractive Proteomics and Reverse Vaccinology

The NCBI’s genome database was consulted to retrieve proteomic information for *H. alvei* [24]. A number of procedures were employed to refine the proteome and evaluate prospective vaccine candidates. Only, completely sequenced strains of the pathogen were used in the study.

### 2.2. Pre-Selection Stage

Researchers examined the bacterial genome for conserved proteins using pan-genome research [25].

### 2.3. CD-Hit Analysis

It is thought that redundancy of biological action results from proteins expressed by two or more genes, and these proteins are ineffective immunological targets due to their bad conservancy since these proteins are not genetic materials. Vaccines should be made with non-redundant proteins meaning proteins that are not duplicated. The CD-HIT online server was used to anticipate all non-redundant proteins of the pathogen with a sequence similarity threshold of 50%, and all other input values were left at their default settings [26]. CD-HIT has become one of the most popular and commonly used servers for comparing and clustering protein sequences.

### 2.4. Subcellular Localization Phase

With PSORTb 3.0, we examined the organization of the essential proteome at the subcellular level. Because they interact with hosts and disrupt the infectious cycle, proteins located or eliminated from infectious agents are important for vaccine development [27]. The human immune system is capable of identifying these antigenic predictors so that targeted responses can be produced.

### 2.5. Vaccine Candidate’s Prioritization Phase

Pathogenic secretomes and exoproteomes were then filtered at this stage to identify those associated with disease progression and pathogenesis. With a sequence identity of less than 30% and a bit score of greater than 100, a BLASTp search was used to identify proteins in the core virulent factor database (VFDB) [28].

### 2.6. Analysis of Potential Transmembrane Helices

Based on the transmembrane helices present in the selected proteins, only proteins with values 0 or 1 were selected [29]. It is easy during an experimental investigation to purify proteins with a small number of transmembrane helices. HTMMTOP and TMHMM 2.0 were both online tools used for the analysis of potential transmembrane helices [30].

### 2.7. Physiochemical Properties Analysis

ProtParam’s online tool allows users to calculate the physicochemical properties of the selected virulent proteins, including their molecular weight, number of amino acids, theoretical PI grand average of hydropathy, stability index, and aliphatic index [31]. To identify the stable protein, a threshold value was set as 40 and the proteins having an instability index value > 40 are considered unstable proteins which were discarded from the study subsequently. Likewise, those proteins with a molecular weight of less than 110 KDa were thought to be good vaccine targets [32].

### 2.8. Homology Check with Human and Normal Flora

The proteins were then used for homology check against human and human beneficial probiotic bacteria to remove the homologous proteins and extract only non-homologous proteins to ensure that human proteins and beneficial bacteria are not accidentally inhibited. For this, we used a server named BLASTp to search against homo sapiens (tax id: 9606) and *Lactobacillus* species: *Lactobacillus casei* (taxid: 1582), *L. rhamnosus* (taxid: 47715), *L. johnsonii* (taxid: 33959) and with human (taxid: 9606) [28,33]. The search parameters include a sequence identity percentage that should be less than 30% and a bit score ≥ 100. So, it becomes easier to evaluate their in vivo immune protective potential without having to worry about false positives and auto-immune reactions. Once the proteins had been filtered, they were examined in terms of the epitope prediction phase, which predicted B-cell derived T-cell epitopes.

### 2.9. Prediction of Immune Cell Epitopes

Using the Immune Epitope Database (IEDB) with the value of 0.5, Bepipred Linear Epitope Prediction 2.0 was used to predict the first linear B-cell epitope for proteins [34]. Following this, the antigenic determinants in the B-cell markers were used to locate subsequences with interactions with MHC (I and II) alleles in the IEDB T-cell antigenic determinants analysis package [35]. In case of MHC-I epitopes prediction, we use HLA-A*01:01, HLA-A*01:01, HLA-A*02:01, HLA-A*02:01, HLA-A*02:03, LA-A*02:03, HLA-A*02:06, HLA-A*02:06, HLA-A*03:01, HLA-A*03:01, HLA-A*11:01, HLA-A*11:01, HLA-A*23:01, HLA-A*23:01, HLA-A*24:02, HLA-A*24:02, HLA-A*26:01, HLA-A*26:01, HLA-A*30:01, HLA-A*30:01, HLA-A*30:02, HLA-A*30:02, HLA-A*31:01, HLA-A*31:01, HLA-A*32:01, HLA-A*32:01, HLA-A*33:01, HLA-A*33:01, HLA-A*68:01, HLA-A*68:01, HLA-A*68:02, HLA-A*68:02, HLA-B*07:02, HLA-B*07:02, HLA-B*08:01, HLA-B*08:01, HLA-B*15:01, HLA-B*15:01, HLA-B*35:01, HLA-B*35:01, HLA-B*40:01, HLA-B*40:01, HLA-B*44:02, HLA-B*44:02, HLA-B*44:03, HLA-B*44:03, HLA-B*51:01, HLA-B*51:01, HLA-B*53:01, HLA-B*53:01, HLA-B*57:01, HLA-B*57:01, HLA-B*58:01, HLA-B*58:01) while in case of MHC-II alleles; HLA-DRB1*01:01, HLA-DRB1*03: *04:01, HLA-DRB101, HLA DRB1*04:05, HLADRB1*07:01, HLA, DQA1*03:01/DQB1*03:02, HLADQA1*03:01/DQB1*03:02, HLADQA1*01:02/DQB1*06:02, HLADPA1*02:01/DPB1*01:01, HLADPA1*01:03/DPB1*04:01, HLADPA1*03:01/DPB1*04:02, HLADPA1*02:01/DPB1*05:01, HLADPA1*02:01/DPB1*14:01 were used.

### 2.10. MHCPred 2.0 Analysis

To examine binding affinities, MHCPred 2.0 was evaluated using the IC50 values less than 100 nM for DRB*0101 to evaluate screened B-cell produced T-cell antigenic determinants [36].

### 2.11. Antigenicity, Allergenicity, Solubility and Toxicity Prediction

The antigenicity of the proteins was determined using VaxiJen 2.0 [37] and a threshold of >0.4 for bacteria as the target cell. The protein allergenicity of proteins was measured with Allertop 2.0 through the innovagen solubility server, we checked the solubility of the epitopes and selected only those epitopes which have good water solubility [38]. For checking the toxicity of epitopes whether the epitope is a toxin or not we used toxin-pred analysis and discarded toxin epitopes [39].

### 2.12. Multi-Epitopes Peptide Designing

Immunogenicity is a weakness of peptide vaccines that can be overcome by combining immunodominant epitopes with appropriate adjuvants and constructing multiepitope peptide vaccines. Our multi-epitope peptide was composed of filtered epitopes assembled with GPGPG linkers [40]. SCRATCH’s protein predictor was used to simulate the 3D structure of the design. Using Galaxy Loop and Galaxy Refine of Galaxy Web, all loops within the structure have been simulated and optimized [41,42,43]. In the proposed vaccine structure, the disulfide bonds were introduced where required in order to increase the structural stability using Design 2.0 [44].

### 2.13. Codon Optimization

In the design sequence for the vaccine, codon usage has been optimized for *E. coli* variants that are distinct from human strains. Utilizing the Java Codon Adaptation Tool (JCat), the cloned sequence was overexpressed in the expression systems [45]. We used the codon adaptation index (CAI) and percent GC concentration to determine whether cloned sequences are expressed. As a rule of thumb, the CAI should be 1. As a result of the high efficiency of transcription and translation, it is recommended that the GC content be between 30 and 70 percent.

### 2.14. Docking and Refinement

To determine the vaccine construct’s affinity for a certain immune molecule, the recombinant construct was docked with a suitable immune receptor in this phase of development. Based on the blind dock method, the vaccine construct is predicted with a possible binding site and orientation with receptor TLR-4 (PDB ID: 4G8A), MHC-I (PDB ID: 1I1Y) and MHC-II (PDB ID: 1KGO). We performed molecular docking using PATCHDOCK, an online network for synchronizing two molecules based on the concept of shape complementarity [46]. The clustering RMSD was set to 4.0 and the complex type to “default”. The docked complexes were fine-tuned instantly using Fast Interaction Refinement in Molecular Docking [47]. Docking solutions for protein–protein can be made faster and more accurate by rescoring and refining them with a Fire dock. By using intermolecular interaction as well as binding confirmation selection, UCSF Chimera selected a complex with a low global binding energy for each case [48]. Low global binding energy indicated a strong and efficient binding affinity.

### 2.15. Molecular Dynamics (MD) Simulation Assay

The dynamic behavior of vaccine-immune receptors can be studied using a molecular dynamics simulation technique. The best docked complex in each receptor was simulated using AMBER16 simulation software (Developed by University of California, Sans Francisco, CA, USA). The simulation was performed on a time scale of 100 ns [49]. To set up the AMBER simulation environment, a SANDER module was set to perform complex preprocessing and simulation trajectories production. The simulation protocol was divided into three parts; preprocessing involving topologies generation, heating, equilibrium and a production run of 100 ns. The antechamber program was used for topologies generation while Tleap was employed to record the topologies. The complexes were solvated into a TIP3P solvation box, and the padding distance between the complex and water box was set at 12 Å. Afterward, the gradual heating of the complexes to 300 K for 1 s was performed. Moving ahead, the complexes were equilibrated for 1 s. The trajectory files were recorded at a rate of 10 ns per production file. For temperature control, the Langevin dynamic was used, while the SHAKE method was used to restrict hydrogen bonds. The electrostatic interactions were modeled using the particle-mesh-Ewald method. The cut-off value set for non-bounded interaction was 8.0 Å. The simulation trajectories were evaluated using the CPPTRAJ module of AMBER

### 2.16. Free Energy of TLR4 and Vaccine Design

The binding free energies were calculated using the AMBER16 tool MMPBSA.py for both TLR4 and multi-epitope vaccine designs [50]. The binding energy was determined for 100 frames from a simulation of the entire trajectory. The key objective of this study was to determine the difference between a solvated state and an unsolvated state in terms of free energy.

## 3. Results

### 3.1. Genomes Retrieval of H. alvei

The NCBI database is necessary for retrieving genome sequences for the development of vaccines based on many epitopes [24]. we download eleven genome sequences for *H. alvei* both complete and incomplete. These pathogens have strain sizes ranging from 4.50 Mb to 4.77 Mb, while their GC content is from 48.70 to 49.00. Table 1 provides information about the type, genome size, and GC content of a strain.

### 3.2. Bacterial Pan-Genome Analysis

**Figure 2:**
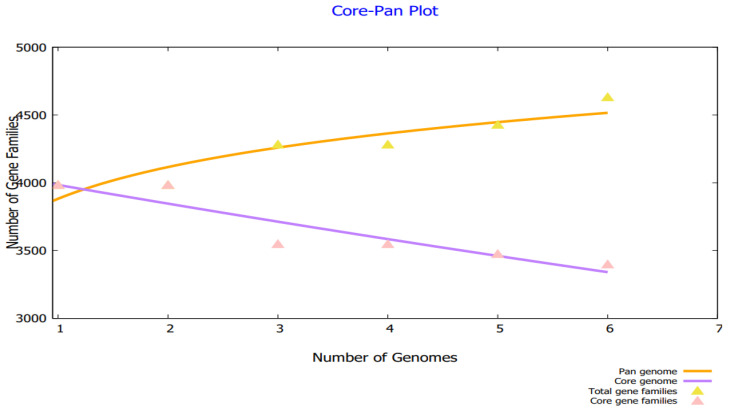
Core pan plot of *H. alvei* strains.

**Figure 3:**
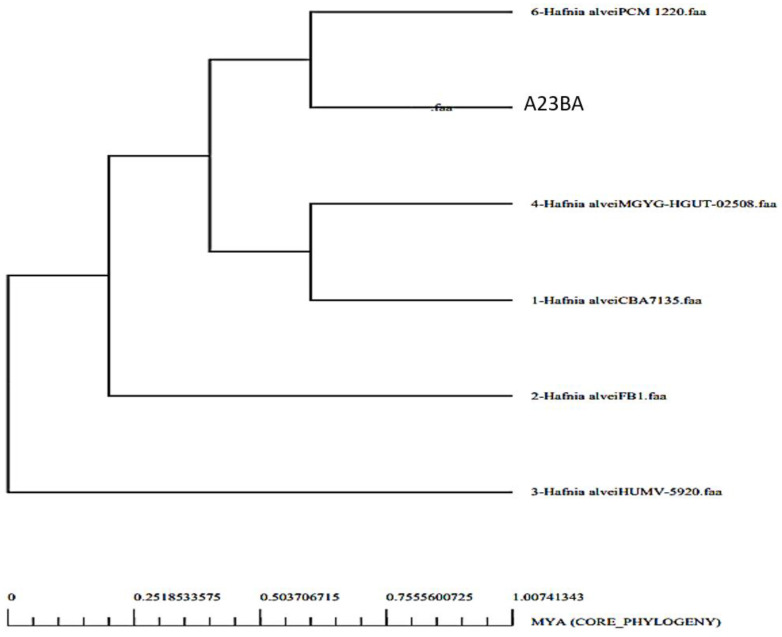
Phylogenetic tree of *H. alvei* strains.

By analyzing the bacterial genome, we were able to derive the core genome and accessory genome. Core genomes contain the sequences that are present in all strains, whereas pan-genomes include sequences of all strains. Accessories include sequences that occur in a small number of strains but do not exist in all strains. There are strain-specific genes that are unique to only one strain. These are also called singletons. The accessory proteome contains genes that are either adjacent or dispensable, while the core genome contains the proteins that are conserved across strains. The genome size of each strain is shown in Figure 2, and a phylogenetic tree of *H. alvei* is shown in Figure 3. The phylogenetic tree of the pathogen strains is given in Figure 3.

### 3.3. CD-HIT Analysis

**Figure 4:**
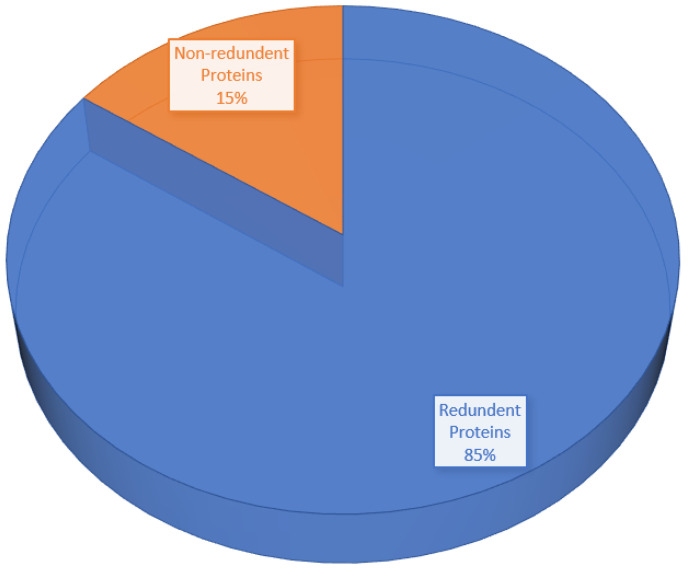
Pie-chart shows the percentage of redundant proteins and non-redundant proteins.

The core genome of the pathogen is comprised of 3111 non-redundant proteins and 17,259 redundant proteins, as shown in Figure 4. Due to their duplicate sequences, the redundant proteins were removed from consideration as vaccine candidates. Further processing was conducted on the non-redundant proteins [26].

### 3.4. Proteins Subcellular Localization

**Figure 5:**
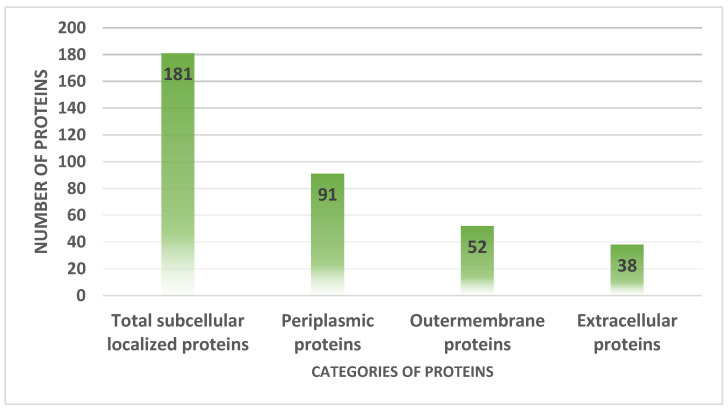
Number of proteins present in extracellular matrix, periplasmic space and extracellular membranes.

The host immune system recognizes the proteins that are present on the surface and in the periplasm, extracellular, and outer membranes [51]. The pathogen surface contained 181 proteins, 91 of which were periplasmic, 52 were outer membrane proteins and 38 were extracellular proteins, as illustrated in Figure 5.

### 3.5. VFDB Analysis

According to the methodology described in the Section 2, 30 virulent proteins were identified. Table 2 shows that in these results, 9 outer membranes, 11 periplasmic, and 10 extracellular proteins were selected. The presence of virulent proteins can act as an attractive vaccine target since they can trigger immune pathways that lead to better immune responses. From the set of exposed proteins, the following virulent proteins were identified.

### 3.6. Transmembrane Helices and Physiochemical Analysis

A protein with 0 or 1 transmembrane helices was selected in the transmembrane helices’ analysis [29]. There were 30 proteins in total in this analysis, so 18 proteins were removed. Thanks to their ease of cloning and expression analysis, such proteins are easy to analyze experimentally. A protein with greater molecular weight was discarded based on physiochemical analysis. Table 3 shows the shortlist of twelve vaccine candidates that fulfill the criteria.

### 3.7. Similarity with Human Genome and Prediction of Antigenicity and Allergenicity

We analyzed these proteins for homology after they have been analyzed for physiochemical analysis. As homologous proteins may cause autoimmune diseases, no homologous proteins should be used in the design of vaccines. In this case, only proteins that were not homologous to the human genome were used. A similar selection was made for proteins that were antigenic and not allergenic as mentioned in Table 4 [52].

### 3.8. Homology Check of Normal Flora

Table 5 also reveals that the selected twelve proteins do not show homology with the normal flora of *Lactobacillus* species; *Lactobacillus casei*, *Lactobacillus jhonsoni*, and *Lactobacillus rhamnosus* are used as bacterial strains. As a result of this analysis, proteins that did not inhibit host normal flora accidentally were selected.

### 3.9. B-Cell Epitopes Prediction

The epitopes for the selected 12 proteins have been predicted after passing through all necessary filters for finding a good vaccine candidate. After that, we will use the IEDB server to predict B-cell epitopes and T-cell epitopes [53]. B-Cell epitopes were predicted first as mentioned in Table 6.

### 3.10. MHC-I and MHC-II Epitopes Prediction

Inferring T-cell epitopes is a multistep process that begins with MHC-I binding and goes on to MHC-II binding. The length of MHC-I and MHC-II alleles interacting with the reference set of MHC alleles and having low percentile scores are given in Table 7. Low percentile score epitopes are strong binders.

### 3.11. Epitope Prioritization Phase

To prioritize those epitopes that can be used to design a multi-epitope vaccine, several filters were applied, such as MHCPred, water-solubility, toxicity, allergenicity, and antigenicity.

### 3.12. MHCPred Analysis

It was determined that epitopes bound to DRB*0101 via MHCPred. A total of 100 epitopes with an IC_50_ value < 100 nM were included in the analysis because they are good binders of DRB*0101, which is the dominant allele present in 95% of the population. The IC50 values of epitopes smaller than 100 nM are listed in Table 8.

### 3.13. Allergenicity and Antigenicity

To elicit strong and safe immune responses, only antigenic and non-allergic epitopes were chosen. The list of antigenic and non-allergic epitopes can be found here in Table 8.

### 3.14. Analysis of Solubility and Toxicity

InvivoGen was used to check the solubility of epitopes and only those that are soluble were selected. The Toxin-Pred method was used to select non-toxic epitopes. You will find epitopes that are not allergenic, non-toxic, or antigenic and have good water solubility in Table 8. Eventually, a multiepitope vaccine will be developed from these selected epitopes.

### 3.15. Multi-Epitopes Vaccine Designing

**Figure 6:**
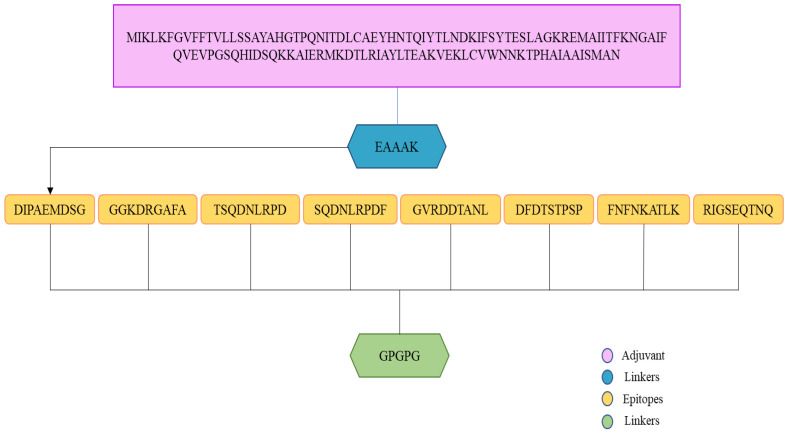
An illustration of the vaccine construct. A light orange color indicates that the epitopes are linked by the GPGPG linker (green color). Adjuvants (cholera toxin B-subunit) and receptors (EAAAK) are represented by pink and blue colors, respectively.

A multi-epitope design was developed in order to improve epitope immunogenicity. To allow efficient separation of the epitopes, linkers were used to join the epitopes together. Furthermore, the vaccine contains an adjuvant molecule to enhance the antigenicity and immunogenicity of the multi-epitope’s peptide. Cholera toxin B-subunit was used as an adjuvant, which is a potent inducer of interferons and cellular immunity. An illustration of a vaccine construct based on multi-epitopes is shown in Figure 6.

### 3.16. Vaccine Structure Modeling

**Figure 7:**
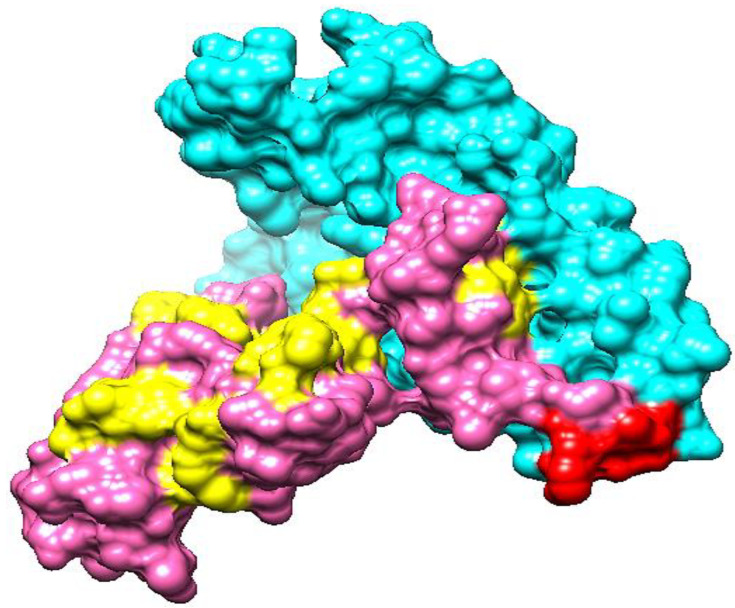
The 3D structure of a candidate vaccine construct. The cyan, red, pink, and yellow colors represent adjuvant, EAAAK linker, epitopes and GPGPG linkers, respectively.

Modeling a three-dimensional structure of the vaccine construct further explained how the vaccine binds with immune receptors and how the vaccine epitopes are exposed. As no template had been available to model vaccine structure, Ab initio structure modeling was performed. A three-dimensional model of the vaccine was developed as shown in Figure 7. The structure validation was conducted using Ramachandran plot analysis. The analysis predicted 90% of residues in Ramachandran plot favored regions, and 1% of residues in Ramachandran disallowed regions.

### 3.17. Loop Modeling and Refinement

Loop remodeling was performed to prevent the formation of structure instability upon loops containing residues; ALA17-GLY21, GLU50-MET58, ALA67-GLU72, GLU100-ASN111, ALA128-MET135, ASP136-PRO142, ASP147-GLY153, PRO154-SER159, GLN160-ASP166, GLY167-GLN173, ASP174-ASP179, PHE180-GLY186, ASP190-PRO196, GLY197-ARG202, ASP203-GLY209, PRO210-PHE215, ASP216-PRO222, GLY223-ASN229, PHE230-LYS236, GLY237-ARG242. In order to obtain the most refined structure, elements of secondary structure were modeled.

### 3.18. Disulfide Engineering

**Figure 8:**
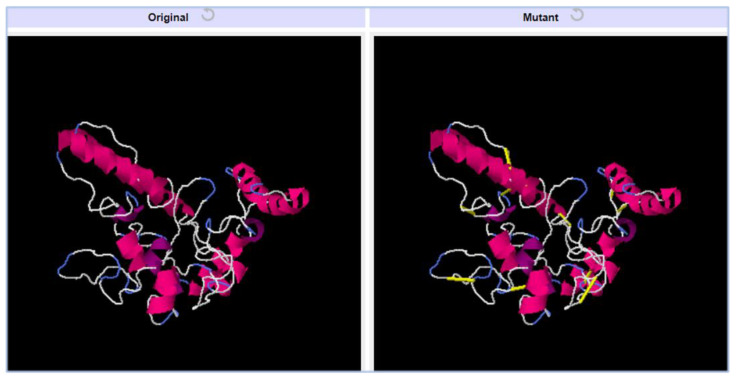
Vaccine constructs with mutated structure. Yellow bands in the mutant structure indicate disulfide bonds were introduced.

Vaccine structure stability and intermolecular bonding were further enhanced through disulfide engineering [54]. Additionally, weaker segments of the vaccine will be resistant to cellular degradation and will retain their conformation when exposed to cellular degradation. We mutated cysteine to just those residue pairs with the highest energy value (over 0 kcal/mol). The yellow sticks indicate cysteine bonds in Figure 8. In total, five pairs of residues were mutated based on the lowest energy score in kcal/mol. The five pairs of residues are; Pro154-Ser159, Gly167-Gln173, Phe180-Gly186, Asp190-Pro196, and Asp216-Pro222.

### 3.19. Codon Optimization

**Figure 9:**
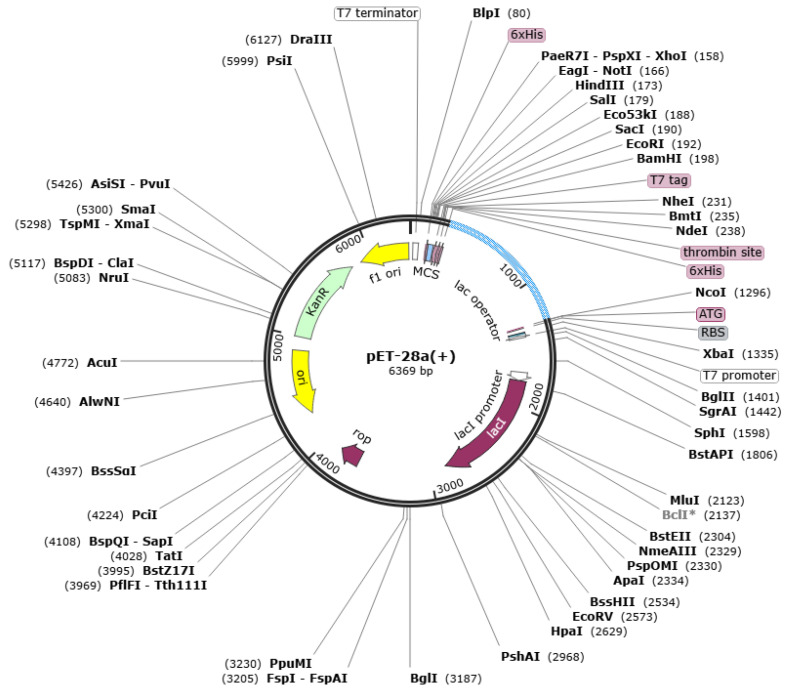
Vector with cloned vaccine. The vaccine is shown by sky blue color while the vector is in black.

To perform its codon optimization according to the *E. coli* expression system, the vaccine sequence was reverse translated into a DNA sequence. Both values, such as CAI (0.98) and GC (56.23%) are indicators of high expression. Additionally, vaccine cloning was conducted in the pET-28a (+) vector as shown in Figure 9.

### 3.20. Analysis of Molecular Docking

For vaccines to generate good immune responses, they must interact strongly with their receptors. We use blind molecular docking to study interactions between host receptors and vaccine constructs. Tables S1–S3 are the top 20 docked vaccine solutions with MHC-I, MHC-II, and TLR-4.

### 3.21. Docked Complexes Refinement

We further refined the docked complexes by removing false positives (docked solutions with high global energies) and selecting the minimal binding energy complex. Immune receptors bind to vaccines with the lowest binding energy complex. For MHC-I, number 5 was selected due to its global energy of −13.83 kJ.mol^−1^, which is the lowest. MHC-II, solution number 2 with −11.10 kJ.mol^−1^ binding energy value was chosen. The lowest global energy is found for TLR-4 with solution number 9 with global energy of −13.10 kJ.mol^−1^. The rescored docked solutions are generated by FireDock in Tables S4–S6. The docked intermolecular conformation of molecules varies significantly among the top solution and the rest.

### 3.22. Docked Confirmation of Vaccine with Immune Receptors

**Figure 10:**
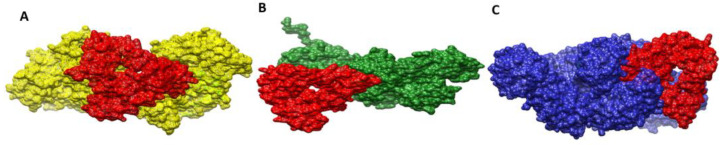
Docked conformation of vaccine (represented by the red mesh) to MHC-I (represented by the yellow mesh) (**A**), MHC-II (represented by the dark green mesh) (**B**), and TLR4 (represented by the blue mesh) (**C**).

To explore how vaccines dock with immune receptors like MHC-I, MHC-II, and TLR-4, the best-docked complex for each receptor has been visualized as shown in Figure 10. There is deep binding between the vaccine and its receptors, exposing the epitopes for recognition and processing by the cells of the host immune system. The formation of strong and protective immune responses is further implied by the fact that vaccine epitopes can stimulate immune pathways.

### 3.23. Interactions of Vaccine to Immune Receptors

In order to accurately determine the effectiveness of vaccine–receptor interactions, it is crucial to understand the type and number of interactions between the vaccine and receptors. Interactions between vaccine and receptor have been observed in a variety of types, including hydrophilic, hydrophobic, salt bridges, and disulfide bonds. The interactions between the vaccine and its immune receptors play a key role in its docked conformation. The receptors engage the vaccine molecules via a number of residues in their structure. These residues can be seen in Table 9. The interaction analysis within 5 Å predicted 18 hydrogen bonds, 231 van der Waals contacts and 200 other hydrophobic contacts between the vaccine and TLR-4. The vaccine molecule interacts with MHC-I via 11, 143 and 147 hydrogens, van der Waals and other hydrophobic interactions, respectively. Similarly, in the case of the vaccine–MHC-II complex, 15 hydrogen bonds, and 176 van der Waals bonds were reported.

### 3.24. Molecular Dynamic Simulation

**Figure 11:**
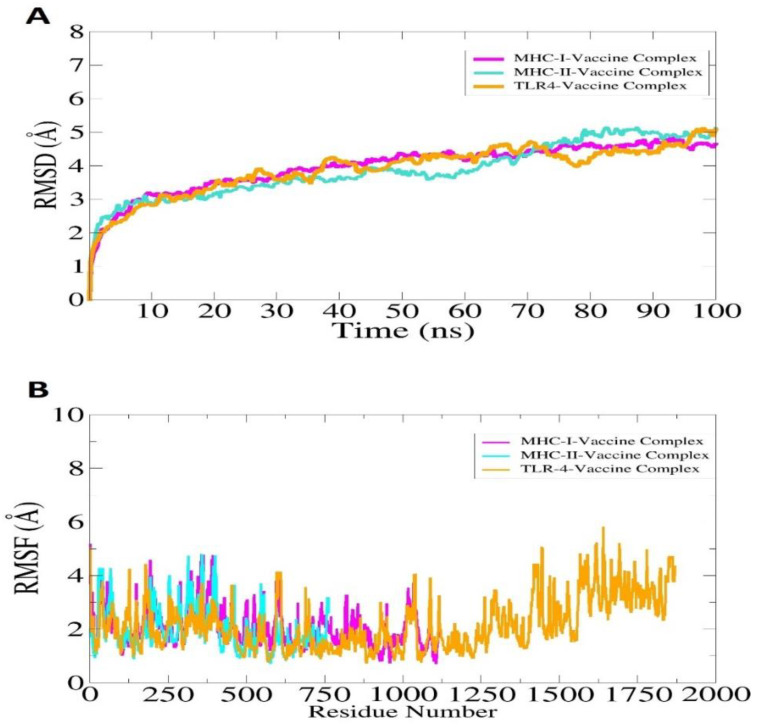
Simulation trajectories analysis of the vaccine-immune receptors. RMSD (**A**) and RMSF (**B**). In the case of RMSF, the TLR-4 length is from Glu1 to Asn1478, MHC-I length is from Gly1 to Met599, and MHC-II is from Glu1 to Ser519. The afterward residues in ease case till the end represent the vaccine molecule.

A molecular dynamics simulation was conducted on selected docked complexes to check their dynamic behavior. On the basis of carbon alpha atoms, root mean square deviations (RMSDs) and root mean square fluctuations (RMSFs) were analyzed for simulation trajectories. It was vital to conduct this analysis to determine whether the epitopes of the vaccine are exposed to the host immune system and how dynamic binding stability relates to receptors. No major changes or conformation deviations were observed in the structures and the plot of RMSD remained stable with very minor curves in the graph at the end. This plot results in a RMSD value of 4.5–5.5 Å during the simulation period, as shown in Figure 11A. The minor RMSD changes correspond to flexible loop dynamics, which do not affect overall intermolecular binding and stability. Furthermore, RMSF indicated that major receptor binding residues remained stable, displaying only a few high degrees of flexibility in the case of vaccine molecules. A majority of the residues in this system are less than 5 Å, which indicates that they have good stability (Figure 11B). In the RMSF plot, the TLR-4 length is from Glu1 to Asn1478, MHC-I length is from Gly1 to Met599, and MHC-II is from Glu1 to Ser519. According to the radius of gyration (RoG), the systems were found compact in nature, and secondary structures were confirmed. As with RMSD and RMSF, these results indicate a fairly stable system.

### 3.25. Calculation of Binding Free Energies

MM-GBSA and MM-PBSA approaches were used to assess the binding free energies of the docked complexes [50]. Despite the modest speed and good accuracy, both of these approaches are considered of high interest in validating docking results. In MM-GBSA, the free binding energy of the vaccine–TLR-4 complex is −123.31 kcal/mol, the vaccine–MHC-I complex is −178.68 kcal/mol and the vaccine–MHC-II complex is −126.63 kcal/mol as mentioned in Table 10. Similarly, the net binding free energy of the vaccine–TLR-4 complex, vaccine–MHC-1 complex, and vaccine–MHC-II complex is −134.05 kcal/mol, −103.57 kcal/mol and −136.92 kcal/mol, respectively. As can be seen in the Table, in both approaches the van der Waals energy dominates overall binding, followed by electrostatic energy.

## 4. Discussion

As a facultatively anaerobic gram-negative bacteria, *H. alvei* belongs to the Enterobacteriaceae family. *H. alvei* is the sole species in the Hafnia genus. The name Hafnia comes from the Latin word hafnia, which means Copenhagen in English [14]. As part of the normal microbiome, Hafnia is found in people’s gastrointestinal tracts and in many animals as well. Despite being rare, *H. alvei* is an extremely poorly understood commensal bacterium. Sludge, animal feces and human feces are a source of *H. alvei. H. alvei* is a gram-negative bacterium rare among pathogenic bacteria because it rarely infects humans [15].

A number of past pandemics have shown that vaccines can effectively prevent infections, saving millions of lives. One example of a successful vaccine is the Spanish flu vaccine and the smallpox vaccine, which saved millions of lives from pandemics. Vaccine development has had a significant effect on tackling many diseases around the world. Despite being used for many decades and being very successful, traditional vaccination technology has several limitations, which have shifted the focus to genome-based vaccines [55]. As bioinformatics has become an increasingly important tool in vaccinology, particularly for those pathogens that cannot be cultured under lab conditions and those whose surfaces undergo continuous genetic changes, the scope of vaccinology has significantly expanded. Reverse vaccinology is genome-based vaccinology and has contributed remarkably to designing multi-epitope vaccines. Due to its recent role in the development of the meningococci vaccine, reverse vaccinology, the opposite of traditional vaccinology, has gained more attention [56].

Twelve vaccine targets were studied in this study; Type 1 fimbrial protein, Flagellar hook length control protein FliK, Flagellar hook associated protein FlgK, Porin OmpA, Curli production assembly/transport protein CsgF, Fimbria/pilus outer membrane usher protein, Flagellar filament capping protein FliD, Fimbria/pilus outer membrane usher protein, Molecular chaperone, TonB-dependent hemoglobin /transferrin/lactoferrin family receptor, Flagellar basal body rod protein FlgF and Flagellar hook-basal body complex protein FliE. These were the identified enzymes that met all the requirements for being a vaccine candidate. This ensures the development of a vaccine that covers a broad range of pathogens. Furthermore, these proteins were confirmed to be present on the surface of the pathogen. The immune system of the host can easily interact with these proteins. Antigenic determinants in these proteins can also stimulate the immune system. In addition, the proteins selected are non-homologous to human proteomes, therefore, making them potential candidates to avoid autoimmune reactions. Additionally, these proteins are antigenic and capable of binding acquired immunity products and activating immune signaling pathways. Further immunoinformatic analyses of the proteins indicate they include antigenic epitopes that are nontoxic, nonallergic and have a strong binding affinity to DRB*0101 alleles. Most human populations carry this allele, which leads to robust and accurate immune responses. Using predicted epitopes, it was possible to design multiepitope vaccines to overcome the limitations of single peptide vaccines. In addition to binding to the MHC-I, MHC-II, and TLR4 immune receptors, the designed vaccine also showed stable conformation with different immune receptors. Upon analysis of intermolecular interactions, it was discovered that multiple hydrophobic and hydrophilic interactions were formed between the vaccine molecules and receptor molecules, forming a stable complex. The candidate vaccine was evaluated to determine if it could stimulate the immune system of the host. Immune responses were observed at all three levels, i.e., primary, secondary and tertiary levels. Furthermore, an increased concentration of interleukin and interferons was detected.

As vaccine development advances, computer-aided vaccine design using genomic information is gaining popularity. In addition to saving time and money, it can generate results in a short period of time [57,58]. According to these findings, the vaccine designed is an excellent candidate for testing in vivo and in vitro. In the past, several efforts have been conducted using computer aided vaccine design strategies. This genome based approach identifies novel epitopes not reported previously. For example, the epitopes identified from the meningococcus genome were not surfaced by experimental techniques and thus led to the successful development of a vaccine [6].

## 5. Conclusions and Limitations

A multi-epitope vaccine against a bacterial pathogen, *Hafnia alvei*, is being pursued in this study. It is being developed with several computer-aided vaccine design approaches, including reverse vaccinology, subtractive proteomics, immunoinformatics, and several biophysical analyses. We predicted vaccine epitopes based on twelve potential vaccine targets; Fimbria/pilus outer membrane usher protein, Type 1 fimbrial protein, Flagellar hook length control protein FliK, Flagellar hook associated protein FlgK, Porin OmpA, Molecular chaperone, Curli production assembly/transport protein CsgF, Fimbria/pilus outer membrane usher protein, Flagellar filament capping protein FliD, TonB-dependent hemoglobin /transferrin/lactoferrin family receptor, Flagellar basal body rod protein FlgF and Flagellar hook-basal body complex protein FliE. A number of criteria were used to prioritize the targets, including but not limited to the protein present in the pathogen’s core proteome, cell surface presence, nonhomologous contact with the host, and probiotic bacteria. Experiments are feasible and possible. Similarly, the vaccine’s antigens and epitopes are non-toxic, non-allergenic, and have a high affinity for binding to B-cells and T-cells. Simulation of the host immune system after vaccination revealed primary, secondary and tertiary immune responses. The findings of all these studies indicated that the vaccine would be a suitable candidate to be tested in vivo for immunity protection. The findings and data of the study may contribute to the development of a vaccine against *H. alvei* more rapidly. Although we were quite diligent throughout the study in terms of our selection criteria, there are still some issues that need to be addressed in future studies. Secondly, the vaccine does not test the order of epitopes for optimal activity. Furthermore, the accuracy of the MHC epitope prediction algorithms has not been extensively tested.

## Figures and Tables

**Table 1:** Different statistics of *H. alvei* strains available at NCBI genome database. Only complete sequenced strains are presented here.

S. No	Organism Name	Strain	Size (Mb)	GC%
1.	*H. alvei*	A23BA	4.77	48.77
2.	*H. alvei*	PCM_1220	4.55	48.90
3.	*H. alvei*	HUMV-5920	4.63	48.70
4.	*H. alvei*	CBA7135	4.50	48.90
5.	*H. alvei*	MGYG-HGUT-02508	4.50	48.90
6.	*H. alvei*	FB1	4.71	49.00

**Table 2:** Virulent proteins with exposed surface topology.

Extracellular Proteins	Bit Score	Sequence Identity
core/462/1/Org1_Gene2554	591 bits	57%
core/770/1/Org1_Gene3263	168 bits	37%
core/845/1/Org1_Gene1577	250 bits	30%
core/847/1/Org1_Gene3025	194 bits	30%
core/935/1/Org1_Gene2294	268 bits	36%
core/979/1/Org1_Gene3259	345 bits	50%
core/1075/1/Org1_Gene1236	464 bits	56%
core/2716/1/Org1_Gene1237	279 bits	61%
core/3338/1/Org1_Gene755	111 bits	39%
core/3797/1/Org1_Gene1966	135 bits	49%
**Outer membrane Proteins**		
core/1/1/Org1_Gene3744	2148 bits	39%
core/120/1/Org1_Gene3603	407 bits	32%
core/149/1/Org1_Gene2889	786 bits	48%
core/243/1/Org1_Gene2268	894 bits	63%
core/303/1/Org1_Gene3723	293 bits	31%
core/969/1/Org1_Gene2163	246 bits	36%
core/1517/1/Org1_Gene912	466 bits	70%
core/2828/1/Org1_Gene1578	204 bits	48%
core/168/3/Org3_Gene3997	438 bits	33%
**Periplasmic proteins**		
core/406/1/Org1_Gene3346	464 bits	46%
core/738/1/Org1_Gene745	243 bits	35%
core/1381/1/Org1_Gene1348	520 bits	78%
core/2485/1/Org1_Gene1235	357 bits	70%
core/2509/1/Org1_Gene738	158 bits	34%
core/2667/1/Org1_Gene3604	147 bits	39%
core/2786/1/Org1_Gene1969	207 bits	50%
core/2972/1/Org1_Gene3596	182 bits	46%
core/3741/1/Org1_Gene572	149 bits	53%
core/3781/1/Org1_Gene1239	230 bits	81%
core/4130/1/Org1_Gene147	129 bits	62%

**Table 3:** Shows us the physicochemical analysis of virulent proteins including the transmembrane helices (TMH), amino acid number, the theoretical index (TI), molecular weight (MW), aliphatic index (AI), instability index (II) and GRAVY.

Vaccine Target				Physiochemical Properties				
Extracellular Proteins	HMMTOP	TMHMM	Amino Acid	M.W	T. PI	I. I	A. I	GRAVY
core/770/1/Org1_Gene3263	0	0	469	48.76661	6.09	39.75	84.48	−0.39
core/845/1/Org1_Gene1577	0	0	456	48.51884	4.4	29.03	95.39	−0.345
core/935/1/Org1_Gene2294	0	0	440	47.62321	4.78	19.79	78.32	−0.512
core/3338/1/Org1_Gene755	0	0	175	18.74466	5.9	20.37	105.26	0.129
core/3797/1/Org1_Gene1966	1	0	136	14.82368	5.15	28.26	83.82	−0.254
Outer membrane proteins								
core/120/1/Org1_Gene3603	1	0	865	95.03412	6.51	33.61	69.69	−0.501
core/149/1/Org1_Gene2889	1	0	824	91.28249	5.55	36.3	70.91	−0.437
core/243/1/Org1_Gene2268	1	0	705	76.71293	5.17	25.66	63.12	−0.517
core/1517/1/Org1_Gene912	1	0	351	38.01163	5.73	26.46	78.15	−0.341
Periplasmic proteins								
core/2485/1/Org1_Gene1235	0	0	251	26.78204	4.74	29.41	85.22	−0.294
core/2667/1/Org1_Gene3604	0	0	237	26.36229	9.32	36.11	82.24	−0.201
core/4130/1/Org1_Gene147	0	0	104	11.07165	5.14	32.46	98.56	0.018

**Table 4:** Lists the proteins that are non-similar, antigenic, and non-allergic to hosts.

Extracellular Proteins	Antigenicity	Allergenicity	Human (taxid:9606)
core/770/1/Org1_Gene3263			
core/845/1/Org1_Gene1577			
core/935/1/Org1_Gene2294	Antigen	Non-Allergen	No Similarity
core/3338/1/Org1_Gene755			
core/3797/1/Org1_Gene1966			
**Outer membrane Proteins**			
core/120/1/Org1_Gene3603			
core/149/1/Org1_Gene2889	Antigen	Non-Allergen	No Similarity
core/243/1/Org1_Gene2268			
core/1517/1/Org1_Gene912			
**Periplasmic Proteins**			
core/2485/1/Org1_Gene1235			
core/2667/1/Org1_Gene3604	Antigen	Non-Allergen	No Similarity
core/4130/1/Org1_Gene147			

**Table 5:** Analysis of homology checks against probiotic bacterial targets.

Extracellular Proteins	*Lactobacillus casei* (taxid:1582)	*Lactobacillus jhonsoni (taxid:33959)*	*Lactobacillus rhamnosus (taxid:47715)*
core/770/1/Org1_Gene3263			
core/845/1/Org1_Gene1577			
core/935/1/Org1_Gene2294			
core/3338/1/Org1_Gene755			
core/3797/1/Org1_Gene1966			
**Outer membrane Proteins**			
core/120/1/Org1_Gene3603		No Similarity	
core/149/1/Org1_Gene2889			
core/243/1/Org1_Gene2268			
core/1517/1/Org1_Gene912			
**Periplasmic Proteins**			
core/2485/1/Org1_Gene1235			
core/2667/1/Org1_Gene3604			
core/4130/1/Org1_Gene147			

**Table 6:** Shortlisted vaccine targets and predicted B-cell epitopes.

B-Cell Epitopes	Peptides
core/770/1/Org1_Gene3263 Flagellar hook length control protein FliK	GSEAQPQWNGSQQNASDRQATSGGFSVD GNNDDRIVTASVSAKSVRVGGV
core/845/1/Org1_Gene1577 Flagellar hook associated protein FlgK	SKANYYDSGQKYIG
	QKIAELEATGGNTNVLRDQRDELVKQMS
	QPDGSQLLTLKYAGSEYSINPATGGQLGA LNDYEQG
	QNQRDNLSAVDQDEE
core/935/1/Org1_Gene2294 Flagellar filament capping protein FliD	QAALKKQQTSLTGQQD
	LNKTNNGLLTNKVTTSLD
	VAFDDMTDDAVNNATG
	NKTNDGSEKSPA
	SGDIPAEMDSGKKTTISEAK
	KLTASGSGGKDRGAFAGDAG
core/3338/1/Org1_Gene755 Type 1 fimbrial protein	TLPVSELARTGQGPEK
	CELTSQDNLRPDFKSVHL
	FDGVRDDTANLLSIHGEAS
core/3797/1/Org1_Gene1966 Curli production assembly/transport protein CsgF	AQNSYKDPNGYDFDTSTPSPLDN
core/120/1/Org1_Gene3603 Fimbria/pilus outer membrane usher protein	FLGGGFAKGLKRFNTDNNTAT
	LTRSPRDAVPVESWDAG
	QSRYRTGSGGTSQY
	GERANKKQGSNNVFKSDTLNQRN
	YQQNRENQAGSTKNWG
core/149/1/Org1_Gene2889 Fimbria/pilus outer membrane usher protein	DDLVEFNTDVLDASDRTHV
	LALKEEARLKVEQVSENCFVLQ
	EYQTSYYNSTHQFDF
	VPAGPFNIQDLSSSVR
core/243/1/Org1_Gene2268 TonB-dependent hemoglobin /transferrin/lactoferrin family receptor	RDRGNLRMSNGFDSPNDET
	INASPTGSSYEKRKQTTNG
	KLENRSRLFADSFAS
	KQKQTPGGATTGFPQAD
	KGSSDGYDDVNADKW
	VTMDMGFVNGRFGCIDCS
	KDQKTGEWLDNINP
	FADRNNQVNAGTAPQA
	EYYTSQGVIQDGI
core/1517/1/Org1_Gene912 Porin OmpA	SHYYDNSINHFGSTNVRPDQLGG
	DWLGRMEYRGNNNGAFKS
	DGSANSETRGRYIDSHDTGVSP
	FNFNKATLKPQGQQA
	RIGSEQYNQKLSEQ
core/2485/1/Org1_Gene1235 Flagellar basal body rod protein FlgF	QLTSQGNLVIGDNGPIAIPDRAEVT
core/2667/1/Org1_Gene3604 Molecular chaperone	ENKVTQLGNKMV
core/4130/1/Org1_Gene147 Flagellar hook-basal body complex protein FliE	LQQLQATAVSAANRSQNSEAPQGA
	AQNFEMGVPGVAL

**Table 7:** From B-Cell epitopes, predictions of MHC-I and MHC-II epitopes.

MHC-I	Percentile Score	MHC-II	Percentile Score
RIVTASVSAK	0.1	DRIVTASVSAKSVRVG	0.73
SVSAKSVRV	0.13		
QATSGGFSV	0.56	DRQATSGGFSVDGN	17
SEAQPQWNG	0.64	GSEAQPQWNGSQQNAS	46
QWNGSQQNA	8.1		
AYYDSGQKY	0.04	SKANYYDSGQKYI	7
EATGGNTNV	0.1	QKIAELEATGGNTNVL	24
KIAELEATG	15		
VLRDQRDEL	0.31	VLRDQRDELVKQMS	3.5
QRDELVKQM	1.7		
SEYSINPAT	0.47	AGSEYSINPATGGQL	4
SINPATGGQL	2.1		
GSQLLTLKY	0.06	QPDGSQLLTLKYAG	13
GQLGALNDY	0.11	PATGGQLGALNDYEQG	17
ATGGQLGAL	3.3		
RDNLSAVDQ	26	RDNLSAVDQDE	0.88
ALKKQQTSL	0.02	AALKKQQTSLTG	1.3
LTNKVTTSL	0.19	GLLTNKVTTSL	0.7
KTNNGLLTNK	0.02	LNKTNNGLLTNKVTTSL	5.3
LTNKVTTSL	0.15		
FDDMTDDAV	5.5	VAFDDMTDDAVNN	21
KTNDGSEKS	5.4	NKTNDGSEKSPA	59
DIPAEMDSGK	1.7	SGDIPAEMDSGK	6.5
EMDSGKKTTI	1.8	EMDSGKKTTISEAK	34
KKTTISEAK	3.8		
KLTASGSGGK	0.23	KLTASGSGGKDRGAFA	21
GSGGKDRGAF	5		
GGKDRGAFA	2.8	GSGGKDRGAFAGDAG	31
KDRGAFAGDA	20		
TLPVSELAR	1.2	TLPVSELARTGQGP	31
SELARTGQGP	2.5		
NLRPDFKSV	0.1	DNLRPDFKSVH	1.8
TSQDNLRPDF	2.5	ELTSQDNLRPDFKSVH	8.1
GVRDDTANL	0.53	DGVRDDTANLL	1.2
DTANLLSIH	0.24	DGVRDDTANLLSIHG	3.6
DGVRDDTANL	1.2		
SYKDPNGYDF	0.05	AQNSYKDPNGYDF	14
DFDTSTPSPL	1.3	NGYDFDTSTPSPLDN	6.1
GFAKGLKRF	0.29	GFAKGLKRFNTDNNTAT	1.1
RFNTDNNTA	3.5		
RDAVPVESW	0.13	RSPRDAVPVESWDA	11
RYRTGSGGT	2.7	RYRTGSGGTSQ	4.7
VFKSDTLNQR	0.16	VFKSDTLNQRN	1.2
KKQGSNNVFK	1.4	RANKKQGSNNVFKSDTLNQR	3.7
NQAGSTKNW	0.25	QQNRENQAGSTKNW	37
DLVEFNTDV	1.1	DDLVEFNTDVLDAS	3.9
VEFNTDVLDA	3.5		
NTDVLDASDR	1.4	LVEFNTDVLDASDRT	4.3
LVEFNTDVL	2.7		
ALKEEARLK	0.11	LALKEEARLKVE	3.1
VEQVSENCF	0.29	ARLKVEQVSENCFVLQ	20
RLKVEQVSE	2.6		
TSYYNSTHQF	0.01	QTSYYNSTHQFDF	6.2
NIQDLSSSVR	0.62	GPFNIQDLSSSVR	6.5
RGNLRMSNGF	6.3	RGNLRMSNGFDSP	5.5
NASPTGSSY	0.01	INASPTGSSYEKRKQTTNG	13
SYEKRKQTT	2.4		
RSRLFADSF	0.21	NRSRLFADSFA	3.3
QTPGGATTGF	0.38	QKQTPGGATTGFP	5.7
SSDGYDDVNA	4.5	GSSDGYDDVNA	6.2
FVNGRFGCI	1.2	DMGFVNGRFGCI	3.3
KTGEWLDNI	0.82	DQKTGEWLDNIN	5.3
QVNAGTAPQA	2.4	FADRNNQVNAGTAPQA	8.1
FADRNNQVNA	3.3		
EYYTSQGVI	0.78	EYYTSQGVIQD	7
SINHFGSTNV	1.1	NSINHFGSTNVR	1.3
EYRGNNNGAF	0.92	WLGRMEYRGNNNGAFKS	1.1
WLGRMEYRG	48		
SANSETRGRY	0.18	SANSETRGRYID	7.6
YIDSHDTGV	0.11	RGRYIDSHDTGVSP	29
RGRYIDSHDT	17		
FNFNKATLK	1	FNFNKATLKPQ	0.85
RIGSEQYNQK	0.2	RIGSEQYNQKLSE	46
DNGPIAIPDR	1.2	NLVIGDNGPIAIPDRAE	2.9
VIGDNGPIAI	2.4		
LTSQGNLVI	1.1	QLTSQGNLVIGDNGPIAI	1.7
IGDNGPIAI	2.1		
KVTQLGNKM	0.7	NKVTQLGNKMV	2.7
ATAVSAANR	0.15	LQQLQATAVSAANRSQNSEAPQGA	0.33
LQQLQATAV	1.1		
FEMGVPGVAL	0.05	AQNFEMGVPGVAL	0.15

**Table 8:** After analysis of MHC-Pred, antigenicity, allergenicity, solubility, and toxin-pred, the following epitopes are shortlisted.

MHC-Pred	MHC-Pred IC_50_ Value (nM)	Antigenicity	Allergenicity	Solubility	Toxin Pred
DIPAEMDSG	84.72				
GGKDRGAFA	95.28				
TSQDNLRPD	9.48				
SQDNLRPDF	16.48	Antigen	Antigen	Good water solubility	Non-Toxin
GVRDDTANL	18.49				
DFDTSTPSP	20.75				
FNFNKATLK	4.44				
RIGSEQYNQ	24.72				

**Table 9:** Vaccine molecules interact with receptor residues.

Vaccine Complex	Interactive Residues
MHC-I	Ala15, Asp 39, Arg181, Asp76, Glu89, Gln98, Glu44, Gly237, His13, Leu130, Lys3, Pro5, Phe56, Ser52, Trp60, Thr73, Val85.
MHC-II	Asp17, Asn78, Arg100, Asp181, Ala140, Glu4, Gly125, Gln311, His143, Leu147, Lys 122, Phe137, Pro127, Ser221, Thr80, Val97.
TLR-4	Arg69, Asn213, Asp50, Cys29, Glu135, Gly110, Gln41, Ile66, Ile285, Leu109, Met40, Met414, Pro53, Ser368, Ser98, Tyr191.

**Table 10:** Different binding free energies between vaccine and receptors. All values are given in kcal/mol.

Energy Parameter	TLR-4-Vaccine Complex	MHC-I-Vaccine Complex	MHC-II-Vaccine Complex
MM-GBSA			
VDWAALS	−115.36	−87.30	−110.00
EEL	−50.00	−53.60	−61.96
EGB	53.54	50.00	55.33
ESURF	−11.49	−12.22	−10.00
Delta G gas	−165.36	−140.9	−171.96
Delta G solv	42.05	37.78	45.33
Delta Total	−123.31	−178.68	−126.63
MM-PBSA			
VDWAALS	−115.36	−87.30	−110.00
EEL	−50.00	−53.60	−61.96
EPB	46.31	48.00	43.10
ENPOLAR	−15.00	−10.67	−8.06
Delta G gas	−165.36	−140.9	−171.96
Delta G solv	31.31	37.33	35.04
Delta Total	−134.05	−103.57	−136.92

## Data Availability

The data presented in this study are available within the article.

## Supplementary Materials

The following supporting information can be downloaded at: https://www.mdpi.com/article/10.3390/vaccines10071127/s1, Table S1: Top 20 vaccine solutions docked to MHC-I. ACE (Atomic contact energy); Table S2: Top 20 vaccine solutions docked to MHC-II. ACE (Atomic contact energy); Table S3: Top 20 vaccine solutions docked to TLR-4. ACE (atomic contact energy); Table S4: Each term given is expressed in terms of energy in kJ.mol^−1^ for FireDock solutions of MHC-I-vaccine. VdW (van der Waals), ACE (Atomic contact energy), HB (hydrogen bonds); Table S5: Each term given is expressed in terms of energy in kJ.mol^−1^ for FireDock solutions of MHC-II-vaccine. VdW (van der Waals), ACE (Atomic contact energy), HB (hydrogen bonds); Table S6: Each term given is expressed in terms of energy in kJ.mol^−1^ for FireDock solutions of TLR4-vaccine. VdW (van der Waals), ACE (Atomic contact energy), HB (hydrogen bonds).
