# Supplementary material for: An In-Silico Investigation to Design a Multi-Epitopes Vaccine against Multi-Drug Resistant Hafnia alvei
Source: Vaccines (Basel). 2022 Jul 15;10(7):1127. doi: 10.3390/vaccines10071127 (PMC9316606; doi:10.3390/vaccines10071127)
Supplement: Supplementary file 1 [file vaccines-10-01127-s001.zip › vaccines-1705220-supplementary.pdf]

Supplementary Files

An *in-silico* investigation to design a multi-epitopes vaccine against multi-drug resistant *Hafnia alvei*

Fahad M. Alshabrmi [1], Faris Alrumaihi [1], Sahar Falah Alrasheedi [1,2], Wafa Abdullah I. Al-Megrin [3,*], Ahmad Almatroudi [1] and Khaled S. Allemailem [1,*]

[1] Department of Medical Laboratories, College of Applied Medical Sciences, Qassim University, Buraydah 51452, Saudi Arabia; fshbrmy@qu.edu.sa (F.M.A.); f_alrumaihi@qu.edu.sa (F.A.); aamtrody@qu.edu.sa (A.A.); k.allemailem@qu.edu.sa (K.S.A.)
[2] Department of Laboratory and Blood Bank, King Saud Hospital, Unaizah 56437, Saudi Arabia; Sahar-f-r@hotmail.com
[3] Department of Biology, College of Science, Princess Nourah bint Abdulrahman University, P.O. Box 84428, Riyadh 11671, Saudi Arabia; Waalmegrin@pnu.edu.sa
* Correspondence: k.allemailem@qu.edu.sa (K.S.A.); Waalmegrin@pnu.edu.sa (W.A.A.)

Table S1. Top 20 vaccine solutions docked to MHC-I. ACE (Atomic contact energy)

Solution No	Score	Area	ACE	Transformation
1	20084	3776.50	461.02	-0.25 -0.01 -0.38 39.82 -3.09 -3.95
2	18446	2303.10	283.20	-1.59 -0.79 -2.72 11.82 42.86 -9.45
3	18114	3113.50	292.26	-0.42 0.37 1.24 31.20 55.64 -0.41
4	17632	2497.30	336.75	1.91 0.78 -2.47 38.51 12.98 67.82
5	17592	2442.60	393.66	2.86 -0.06 0.56 6.24 40.34 50.02
6	17500	3013.10	266.27	-3.12 0.31 0.52 14.25 37.64 60.94
7	17284	2823.10	184.81	-1.38 0.31 2.91 -0.75 -6.32 -23.96
8	17226	2218.90	307.79	1.10 0.72 -0.57 28.41 20.06 -26.61
9	17092	2281.00	481.60	1.21 -0.72 -2.67 20.94 -20.63 -34.95
10	16886	2367.30	476.56	1.22 -0.83 2.18 -28.10 4.06 14.16
11	16868	2206.30	347.88	0.38 -0.36 1.28 -20.43 -0.69 -53.54
12	16868	3684.00	479.35	1.05 -0.48 -2.91 9.58 -17.26 -35.23
13	16858	2595.90	452.45	1.76 -0.18 -1.69 49.60 -14.20 0.59
14	16768	2428.50	470.11	-1.55 -0.22 -2.34 -16.70 1.71 37.68
15	16730	2555.20	426.08	-2.37 0.74 1.10 -4.53 -37.59 23.66
16	16670	2746.30	458.59	1.46 -0.41 1.66 28.12 38.19 51.79
17	16634	2390.10	199.31	1.60 0.72 -2.22 39.36 8.35 62.66
18	16608	2214.70	481.92	-2.61 0.85 1.53 14.18 34.26 36.52
19	16524	2440.70	17.97	-1.00 -0.60 -2.28 -3.00 59.12 -3.65
20	16478	1941.90	487.86	-2.99 -0.77 2.59

Table S2. Top 20 vaccine solutions docked to MHC-II. ACE (Atomic contact energy)

Solution No	Score	Area	ACE	Transformation
1	19808	2637.70	425.40	2.32 1.05 2.24 111.47 14.97 3.54
2	19138	3205.40	230.51	0.22 -0.38 0.36 97.66 51.25 -33.45
3	18730	2614.40	260.01	3.08 -0.07 -1.12 123.77 71.57 45.71
4	18420	2601.20	249.28	-1.34 -0.10 1.00 92.01 73.22 -3.72
5	18294	3049.40	161.53	2.71 -0.40 1.93 76.45 69.85 29.76
6	18044	2808.70	173.91	2.83 -0.42 2.77 73.84 118.18 21.96

7	17998	2924.70	230.12	-2.81 0.43 2.79 118.18 100.28 7.08
8	17966	3320.70	359.77	0.80 -0.54 -0.88 119.50 99.67 -38.90
9	17798	2515.60	16.63	-1.03 0.19 -0.96 116.27 61.48 -29.90
10	17766	3577.20	58.31	-2.60 0.63 2.62 119.52 99.93 5.10
11	17048	2898.20	499.16	-0.26 0.08 -1.74 87.54 62.22 -41.86
12	16972	3016.20	380.12	-0.94 0.47 2.72 134.88 85.41 10.53
13	16964	2385.90	167.63	-3.09 -0.36 2.68 104.83 52.92 48.92
14	16948	2965.00	230.71	1.04 -0.47 -0.07 104.88 122.37 11.67
15	16834	2437.00	356.15	-2.03 -0.02 0.83 115.24 72.94 38.08
16	16778	3084.00	-19.67	1.39 1.09 -1.68 91.94 74.33 -1.13
17	16742	2953.90	360.87	2.99 -0.26 -0.96 132.21 74.56 37.51
18	16736	2919.90	134.64	-0.00 0.37 2.26 90.66 116.50 -31.01
19	16580	2291.30	212.31	2.63 0.22 2.42 106.77 23.73 25.82
20	16564	2295.80	-85.27	2.74 0.69 2.12 131.97 68.01 15.17

Table S3. Top 20 vaccine solutions docked to TLR-4. ACE (atomic contact energy)

Solution No	Score	Area	ACE	Transformation
1	20888	3342.10	418.98	1.91 -0.70 0.63 -58.09 33.36 -74.40
2	20298	3216.30	329.41	-0.59 -0.86 -1.57 -17.46 11.67 -2.46
3	20164	3596.40	157.69	0.31 0.55 -1.81 0.57 -7.60 -78.17
4	19214	2606.70	369.22	-0.79 -0.90 -1.85 -13.90 10.67 -0.89
5	19068	2983.30	226.64	-1.28 0.90 -1.92 -44.47 -35.40 24.78
6	18594	2912.90	79.04	1.76 -0.15 -2.73 -22.26 6.03 4.09
7	18222	3340.20	-137.70	-0.51 -0.98 2.32 8.59 2.74 -2.70
8	17840	2611.60	414.29	-0.82 -0.56 -2.36 -70.54 15.63 -8.75
9	17686	2860.00	5.63	-1.21 0.15 1.34 15.60 -17.07 11.98
10	17668	2664.00	139.44	0.04 -0.50 -2.67 -3.27 43.53 -54.57
11	17666	2373.40	115.73	1.45 -0.47 -0.26 -29.16 56.27 -77.88
12	17648	2151.70	391.40	0.40 1.35 0.76 -47.84 44.69 -50.57
13	17608	2579.70	205.70	0.23 0.49 1.07 21.38 39.68 -71.07
14	17564	2779.90	273.23	-1.01 -0.02 1.72 23.35 -11.34 -17.24
15	17518	3092.50	-7.98	-2.89 0.13 -2.68 9.06 4.36 4.47
16	17364	2840.10	479.91	1.08 0.64 3.06 -13.98 22.28 -57.60
17	17248	2788.00	407.95	2.95 0.55 0.93 -17.51 39.47 -35.51
18	17182	2654.60	352.77	-1.48 -0.45 -1.51 -44.12 10.69 1.80
19	17122	2861.70	451.74	-2.19 -0.07 0.17 32.69 -24.87 -6.27
20	17068	2456.10	181.44	-1.42 -0.93 -1.90 -9.96 5.79 -2.19

Table S4. Each term given is expressed in terms of energy in kJ.mol^{-1} for FireDock solutions of MHC-I-vaccine. VdW (van der Waals), ACE (Atomic contact energy), HB (hydrogen bonds)

Rank	Solution Number	Global Energy	Attractive VdW	Repulsive VdW	ACE	HB
1	8	-21.50	-41.11	25.75	5.56	-6.63
2	9	-13.40	-30.00	15.55	14.99	-3.02
3	2	-8.11	-36.41	18.00	8.82	-1.38
4	10	2.10	-20.24	9.21	10.72	-2.48
5	6	7.42	-4.85	1.17	1.86	0.00
6	4	8.32	-29.45	53.90	6.04	-4.09
7	3	16.74	-6.96	9.67	2.44	0.00
8	5	24.55	-23.68	14.84	12.49	-0.92

9	7	230.32	-23.02	320.66	-0.92	-2.70
10	1	3200.07	-37.79	4005.54	16.86	-4.39

Table S5. Each term given is expressed in terms of energy in kJ.mol^{-1} for FireDock solutions of MHC-II-vaccine. VdW (van der Waals), ACE (Atomic contact energy), HB (hydrogen bonds)

Rank	Solution Number	Global Energy	Attractive VdW	Repulsive VdW	ACE	HB
1	2	-19.72	-13.46	5.29	-5.89	-1.24
2	5	7.13	-0.13	0.00	0.36	0.00
3	3	8.32	-3.50	0.00	4.02	0.00
4	4	30.90	-7.58	18.23	8.47	-0.55
5	8	59.59	-5.74	64.58	4.50	-0.72
6	1	85.77	-25.12	106.46	1.23	-1.19
7	6	192.17	-17.52	256.10	6.96	-3.22
8	7	245.06	-10.66	282.87	4.99	-0.22
9	9	639.37	-45.52	865.45	4.31	-1.39
10	10	1065.77	-53.07	1420.49	9.90	-7.76

Table S6. Each term given is expressed in terms of energy in kJ.mol^{-1} for FireDock solutions of TLR4-vaccine. VdW (van der Waals), ACE (Atomic contact energy), HB (hydrogen bonds)

Rank	Solution Number	Global Energy	Attractive VdW	Repulsive VdW	ACE	HB
1	2	-27.78	-43.87	58.42	13.27	-6.89
2	3	-10.35	-10.11	2.50	1.76	-0.96
3	4	-3.28	-56.17	98.25	16.51	-8.00
4	10	6.15	-13.73	4.41	4.57	-4.59
5	7	6.78	-7.23	5.87	-0.41	-0.86
6	8	9.40	-34.10	18.67	19.01	-6.26
7	9	15.77	-4.56	0.66	4.46	0.00
8	1	18.88	-12.01	16.40	10.21	-1.53
9	5	35.80	-23.87	13.38	17.59	-3.81
10	6	483.78	-43.86	697.80	-0.47	-2.85
